# Overexpression of the *Eucommia* *ulmoides* Aquaporin, *EuPIP1;1*, Promotes Leaf Growth, Flowering and Bolting, and Stress Tolerance in *Arabidopsis*

**DOI:** 10.3390/ijms231911794

**Published:** 2022-10-04

**Authors:** Jiajia Chen, Yanhui Huang, Jianrong Li, Yan Li, Xiaofang Zeng, Degang Zhao

**Affiliations:** 1 Key Laboratory of Plant Resource Conservation and Germplasm Innovation in Mountainous Region (Ministry of Education), College of Life Sciences/Institute of Agro-bioengineering, Guizhou University, Guiyang 550025, China; 2 Guizhou Plant Conservation Technology Center, Guizhou Key Laboratory of Agricultural Biotechnology, Guizhou Academy of Agricultural Sciences, Guiyang 550006, China

**Keywords:** *Eucommia ulmoides*, *EuPIP1;1*, leaf development, bolting and flowering, salt resistance, drought resistance

## Abstract

Plasma membrane intrinsic protein (PIP) is one of the largest subfamilies of Aquaporins (*AQPs*) and plays an important role in plant growth and development, and resistance to abiotic stress. In this study, the full length of the *EuPIP1;1* cDNA was cloned from *Eucommia ulmoides* using the rapid amplification of cDNA ends (RACE) method. The *EuPIP1;1* gene was induced by drought treatment and expressed in all tested tissues, with the highest expression level in fruit. The subcellular localization showed that *EuPIP1;1* was located in the plasma membrane. Constitutive overexpression of *EuPIP1;1* in *Arabidopsis* *thaliana* could promote leaf growth and development, and accelerate bolting and flowering. Six genes related to growth and flowering (*AtPIF4*, *AtTCP14*, *AtCRY1*, *AtCRY2*, *AtFCA* and *AtFT*) were significantly up-regulated in transgenic lines. Further, *EuPIP1;1* gene improved resistance to drought and salt stress in transgenic *Arabidopsis*. Under drought and salt stress treatment, the transgenic lines had a higher germination rate and accumulation of osmotic substances, lower membrane damage, and could maintain ion homeostasis. Our results suggest that *EuPIP1;1* plays an essential role in plant growth and development and in the response to drought and salt stress.

## 1. Introduction

The global shortage of fresh water is one of our most severe ecological and agronomical problems [1]. Water scarcity can lead to land drying and salinization, which reduce plant growth and crop yields [2]. *AQPs* are thought to be the primary transporters of water, as well as small and uncharged solutes, through plant cell membranes [3]. Thus, *AQPs* play an important role in maintaining the balance of cellular water in plants [4]. *AQPs* are usually formed by an assembly of four monomers, each with six transmembrane spanning domains (H1-H6) and five connecting loops (LA-LE) located on the intra- (LB, LD) or extra-cellular (LA, LC, LE) sides of the membrane [5]. The LB and LE loops each carry a highly conserved asparagine-proline-alanine (NPA) motif, folding as half-membrane-spanning α-helices [6]. Plants can dynamically regulate plant water content and transport of small neutral molecules by regulating the abundance and activity of *AQPs*, thereby improving tolerance to external stresses [7]. Several studies have shown that *AQPs* not only play a vital role in regulating plant water balance and mineral nutrient transport [8], but also participate in leaf and petal expansion [9], fruit ripening [10], carbon and nitrogen fixation [11], and signaling [3].

The PIP subfamily is one of the largest subfamilies of *AQP* proteins [12]. This subfamily can be subdivided into two groups, PIP1s and PIP2s [13], which are different in the length of the N- and C- termini and in the conductivity of water [14]. Studies have demonstrated that PIPs play an essential role in seed germination [15], root elongation [16], stomatal movement [17], fruit maturation [18], and response to environmental stress [3]. It has been suggested that under drought stress, *PIPs* may control isohydric/anisohydric behavior and root hydraulics and recovery from that stress [3]. *BjPIP1* enhanced the drought resistance of tobacco by decreasing transpiration via reducing stomatal conductance [19]. Overexpression of *OsPIP2;4* and *OsPIP2;7* in *Arabidopsis* imparted greater tolerance under boron toxicity [20]. The overexpression of *SlPIP2;1*, *SlPIP2;7* or *SlPIP2;5* in transgenic *Arabidopsis* and tomato facilitated significantly higher survival rates under drought stress [21]. Overexpression of *BnPIP1* in tobacco resulted in increased tolerance to water stress, and germination of antisense line seeds was inhibited [22].

*E. ulmoides* is an important afforestation tree species in the construction of ecological forests in China, widely distributed in various regions, with biological characteristics such as hardiness, resistance to drought and strong adaptability [23]. At present, research on *E. ulmoides* has mainly focused on its chemical constituents and their pharmacological effects [24], the gum-containing properties of *E. ulmoides* [25], and the denaturation and modification of the gums of *E. ulmoides* [26]. With the deepening of global drought, water shortages and soil salinization [2], the function of *AQPs* in plants and the response mechanism to drought stress have become one of the hotspot topics. A previous study by Wuyun, et al. [27] showed that 46 *AQPs* were identified from the *E. ulmoides* genome, including the *PIP1* gene family (3), the *PIP2* gene family (17), the *SIP* gene family (5), the *TIP* gene family (11) and the *NIP* gene family (10).

In our previous study, we identified an EST containing a partial gene sequence of the *PIP1* subfamily from the *E. ulmoides* RNA-seq database [28]. Herein, we isolated the full-length sequence of the identified EST (named *EuPIP1;1*), containing a noncoding region of 5′- and 3′-, using the RACE method. The expression pattern of *EuPIP1;1* was identified by qRT-PCR. Furthermore, it was found that overexpression of *EuPIP1;1* could promote growth and improve tolerance to drought and salt of transgenic *Arabidopsis*. We confirmed that *EuPIP1;1* could be used as a functional gene for stress resistance. These results can not only further reveal the function of *AQP* and enrich the response mechanism of *AQP* stress, but also provide new genetic resources for the improvement of tree stress resistance.

## 2. Results

### 2.1. Cloning and Characterization of EuPIP1;1

**Figure 1:**
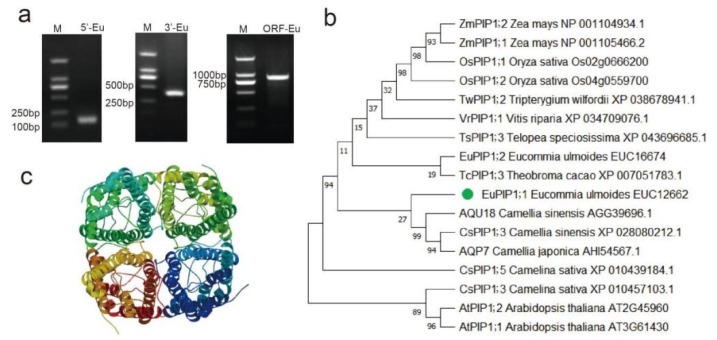
Amplification of *EuPIP1;1* by RACE, phylogenetic analysis and tertiary structure of *EuPIP1;1*. (**a**) Amplification of *EuPIP1;1* by RACE. M: DL2000 marker, 3’-Eu: 3’UTR of *EuPIP1;1*, 5’-Eu: 5’UTR of *EuPIP1;1*, ORF-Eu: the full-length cDNA of *EuPIP1;1*. (**b**) Phylogenetic analysis of *EuPIP1;1* with other PIP proteins. (**c**) Prediction of the tertiary structure of *EuPIP1;1*.

**Figure 2:**
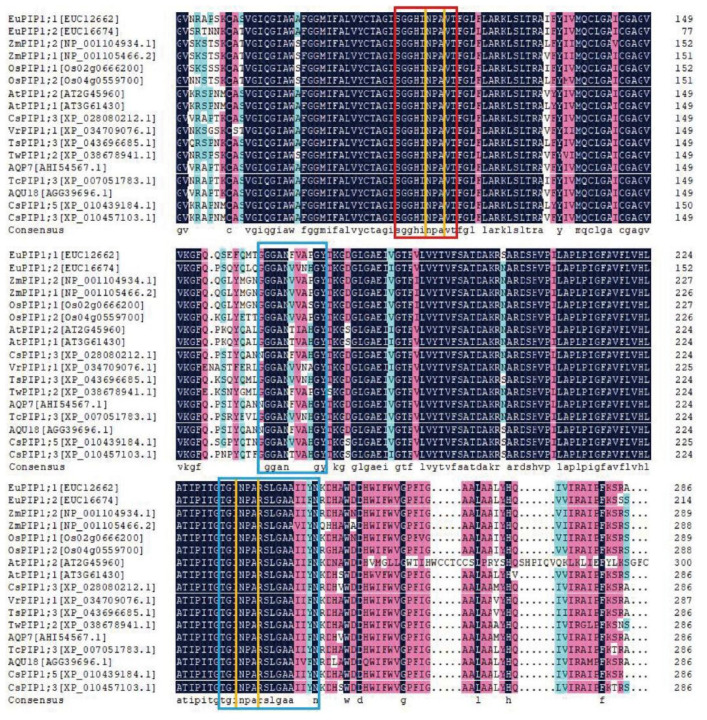
Multiple alignment analysis of *EuPIP1;1* with other species of PIP proteins. The intrinsic protein-specific motif of the MIP family ‘SGGHINPAVT’ is enclosed in the red box, the highly conserved sequences of the PIP of plants ‘GGGANVVNPGY’ and ‘TGINPARSLGAAIIYN’ are indicated in blue boxes, and two ‘NPA’ motifs are enclosed in orange boxes.

Using the RACE method, a full-length of the aquaporin gene cDNA was isolated from *E. ulmoides* and designated as *EuPIP1;1*. The 1130 bp length of the *EuPIP1;1* cDNA sequence contained an open reading frame of 861 bp (encoding 286 amino acids), a 75 bp 5′-noncoding region, and a 166 bp 3′-noncoding region (Figure 1a). The *EuPIP1;1* and EUC12662 [27] cDNA sequences had a synonymous SNP (C-A) at base 752 of the coding region (Figure S1). *EuPIP1;1* contained two conserved ‘NPA’ (Asn-Pro-Ala) motifs (Figure 2, orange box), the intrinsic protein-specific sequence of MIP family (Figure 2, red box), and conserved PIP plant sequences (Figure 2, blue box). Phylogenetic analysis indicated that *EuPIP1;1* was closely related to AQP7 (*Camellia japonica*), AQU18 (*Camellia sinensis*) and CsPIP1;3 (*Camellia sinensis*), and distantly related to rice and maize PIP proteins (Figure 1b). Protein homology-modeling predicted that *EuPIP1;1* could form a homologous tetramer, which had high homology with the *SoPIP2* sequence model (72.80%) (Figure 1c).

### 2.2. Expression Pattern of EuPIP1;1

**Figure 3:**
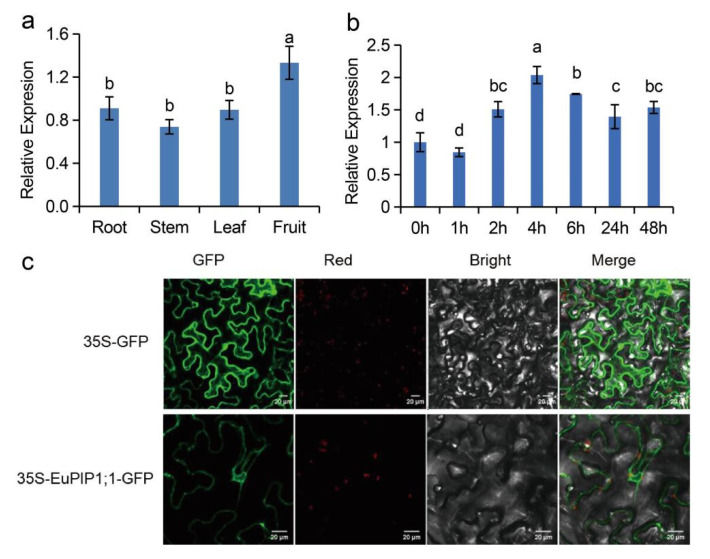
The expression pattern of *EuPIP1;1* and the subcellular localization of the *EuPIP1;1* protein. (**a**) Tissue-specific expression of *EuPIP1;1*. (**b**) Relative transcript levels of *EuPIP1;1* in leaves of *E. ulmoides* treated with 20% PEG6000 for different time. (**c**) Subcellular localization of *EuPIP1;1* in epidermal cells of tobacco leaves. Significant differences were determined by one way ANOVA followed by Duncan’s multiple range test at *p* < 0.05.

Quantitative real-time PCR (qRT-PCR) analysis showed that the *EuPIP1;1* transcript was expressed in the root, stem, leaves, and fruit, with the relative expression level being highest in the fruit, followed by the root, stem, and leaves (Figure 3a). The *EuPIP1;1* transcript level was up-regulated after 2 h PEG treatment and the highest expression level appeared after 4 h (Figure 3b). The result suggested that *EuPIP1;1* was a drought-inducible gene, indicating that *EuPIP1;1* could participate in the response to drought stress.

### 2.3. Subcellular Localization of EuPIP1;1 Protein

To determine the subcellular localization of *EuPIP1;1*, the 35S-EuPIP1;1-GFP construct was transiently expressed in leaves of *Nicotiana benthamiana*. The result showed that the EuPIP1;1 protein was located on the plasma membrane, indicating that the protein belongs to the PIP subfamily (Figure 3c).

### 2.4. Overexpression of EuPIP1;1 Promotes the Growth and Development of Arabidopsis

**Figure 4:**
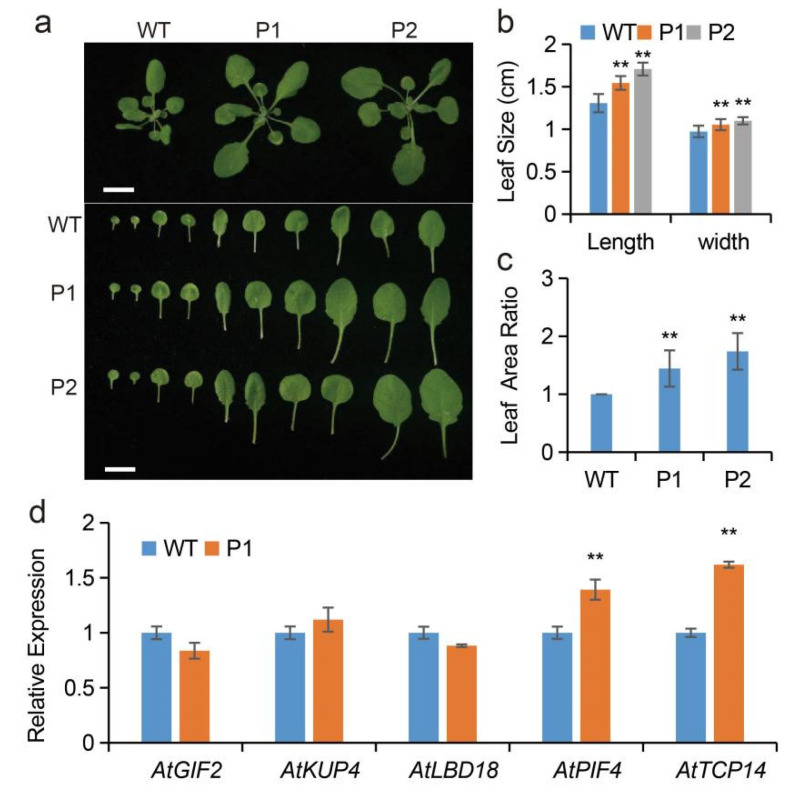
*EuPIP1;1* promotes *Arabidopsis* growth. (**a**) Phenotypes of WT and transgenic lines (P1 and P2) grown in soil for 32 d. Bar = 1 cm. (**b**) The leaf size. (**c**) The leaf area ratio. (**d**) The expression level of growth-related genes in WT and P1. (** *p* < 0.01, one way ANOVA).

**Figure 5:**
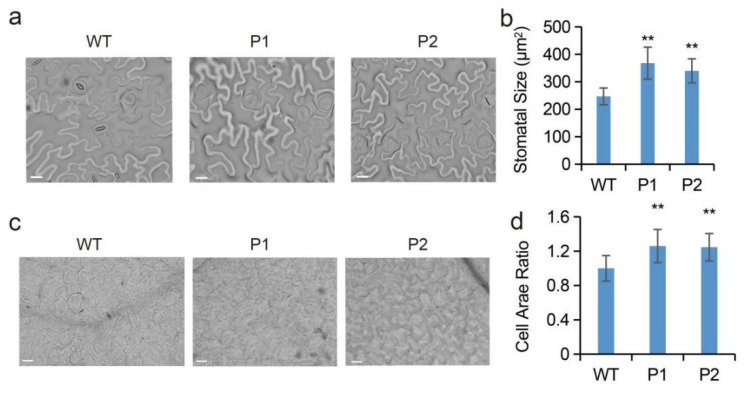
Effect of *EuPIP1;1* on leaf cell size in *Arabidopsis*. (**a**) Stomata distribution in WT and transgenic lines (P1 and P2). Bar = 20 μm. (**b**) The stomatal size. (**c**) Palisade cells of the leaf from WT and transgenic lines (P1 and P2). Bar = 10 μm. (**d**) The cell area ratio. (** *p* < 0.01, one way ANOVA).

To evaluate the function of *EuPIP1;1*, an overexpression vector harboring *EuPIP1;1* was transformed into *Arabidopsis* for heterologous expression. Generation of T_3_ from two transgenic lines P1 and P2 was selected for further analysis. The leaf size of the transgenic lines was significantly larger than that of the WT (Figure 4a,b). The leaf length of WT was 1.30 cm, and the leaf lengths of P1 and P2 were 1.54 and 1.70 cm, respectively (Figure 4b). The width of the leaf of the transgenic lines was significantly wider than that of the WT (Figure 4a,b). By its leaf length, it can be seen that the leaf area of P1 and P2 was 1.44- and 1.74-fold that of the WT (Figure 4c). The qRT-PCR assay showed that two important genes related to growth and development, *AtPIF4* [29] and *AtTCP14* [30], were significantly up-regulated in transgenic plants (Figure 4d). The expression levels of *AtGIF2* [31] (related to leaf growth) and the genes related to root development, *AtKUP4* [32] and *AtLBD18* [33], in transgenic plants were not different from those of WT (Figure 4d). In addition, *EuPIP1;1* could alter stomatal size and cell area in transgenic *Arabidopsis*. The stomatal size in P1 and P2 were significantly increased (49% increase in P1 and 37.7% in P2) ((Figure 5a,b), and the cell areas were 1.26- and 1.25-fold that of the WT, respectively (Figure 5c,d). These results suggested that overexpression of *EuPIP1;1* could promote leaf expansion and could be related to increase in cell size and up-regulation of the expression level of growth-related genes.

**Figure 6:**
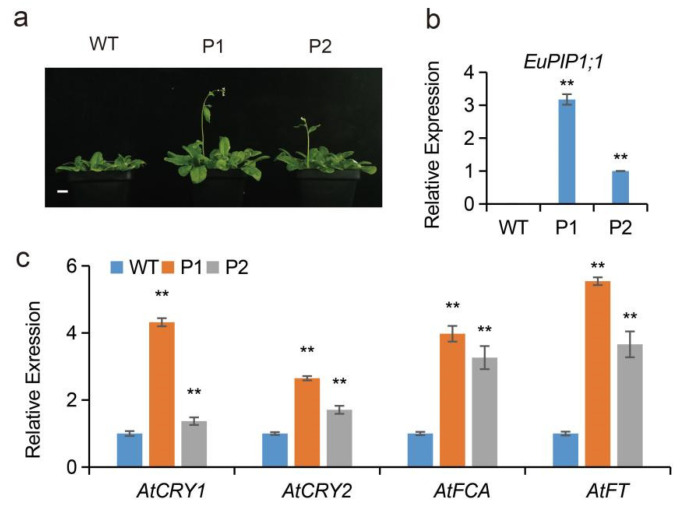
*EuPIP1;1* promotes *Arabidopsis* bolting and flowering. (**a**) Phenotypes of WT and transgenic lines (P1 and P2) grown in soil for 50 d. Bar = 1 cm. (**b**) The expression level of *EuPIP1;1* in WT and transgenic lines (P1 and P2). (**c**) The expression level of flowering-related genes in the leaves of WT and P1. (** *p* < 0.01, one way ANOVA).

Compared to WT, the transgenic *Arabidopsis* showed an early-flowering phenotype (Figure 6a). To test whether the early-flowering phenotype in transgenic lines was related to the up-regulated expression levels of flowering-related genes, the transcriptional levels of four relation genes, *AtCRY1*, *AtCRY2*, *AtFCA* and *AtFT*, were analyzed [34]. The expression levels of these four genes in transgenic plants were elevated to different degrees. The transcriptional levels of *AtCRY1* were 4.31- and 1.37-fold, *AtCRY2* were 2.65- and 1.70-fold, *AtFCA* were 3.98- and 3.26-fold, and *AtFT* were 5.54- and 3.66-fold that of the WT, in the P1 and P2 lines, respectively (Figure 6c). In addition, we examined the expression levels of the *EuPIP1;1* gene in P1 and P2 lines. The results showed that the expression level of *EuPIP1;1* in P1 was 3.17-fold that of P2 (Figure 6b). We demonstrated that overexpression of *EuPIP1;1* could accelerate bolting and flowering might be associated with up-regulated expression levels of flowering-related genes.

### 2.5. Overexpression of EuPIP1;1 Improved Drought and salt Tolerance in Transgenic Arabidopsis

**Figure 7:**
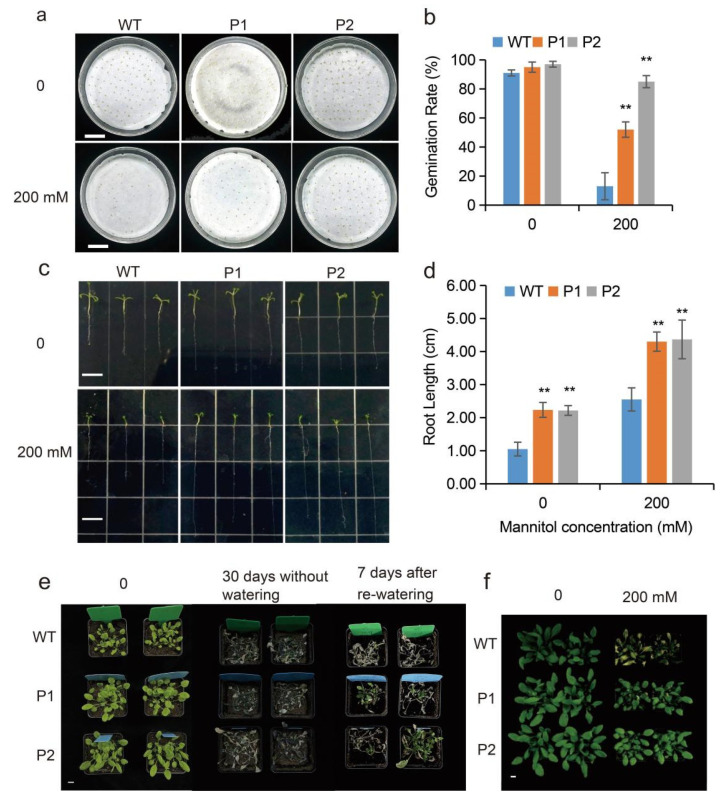
Effect of 200 mM mannitol treatment on germination and root growth of WT and transgenic lines (P1 and P2). (**a**) Phenotypes of WT and transgenic lines (P1 and P2) seeds. Bar = 1 cm. (**b**) The germination rate. (**c**) Phenotypes of WT and transgenic lines (P1 and P2) seedling. Bar = 1 cm. (**d**) Root length. (**e**) Phenotypes of 40-day-old WT and transgenic lines (P1 and P2) under drought stress for 30 d. Bar = 1 cm. (**f**) Phenotypes of WT and transgenic lines (P1 and P2) under 0, or 200 mM mannitol treatment at 11 d. Bar = 1 cm. (** *p* < 0.01, one way ANOVA).

To investigate the effect of *EuPIP1;1* on the tolerance to drought of transgenic *Arabidopsis*, the WT and transgenic *Arabidopsis* (P1 and P2) seeds were germinated on filter paper moistened with 0 or 200 mM mannitol for 7 days. On normal conditions, the germination rates of the transgenic plants for P1 and P2 were not significantly different to those of WT. Under mannitol treatment, the WT germination rate decreased to 13%, while the germination rate of P1 and P2 was significantly higher than that of the WT (52% for P1 and 85% for P2) (Figure 7a,b).

The root length of 17-day-old P1 and P2 seedlings was longer than that of the WT under normal and mannitol-treated conditions (Figure 7c,d). In normal, the root lengths of P1 and P2 were 2.12- and 2.11-fold that of the WT, respectively, while under mannitol treatment, the root length of P1 and P2 plants increased to 1.69- and 1.71-fold that of the WT, respectively (Figure 7c,d).

**Figure 8:**
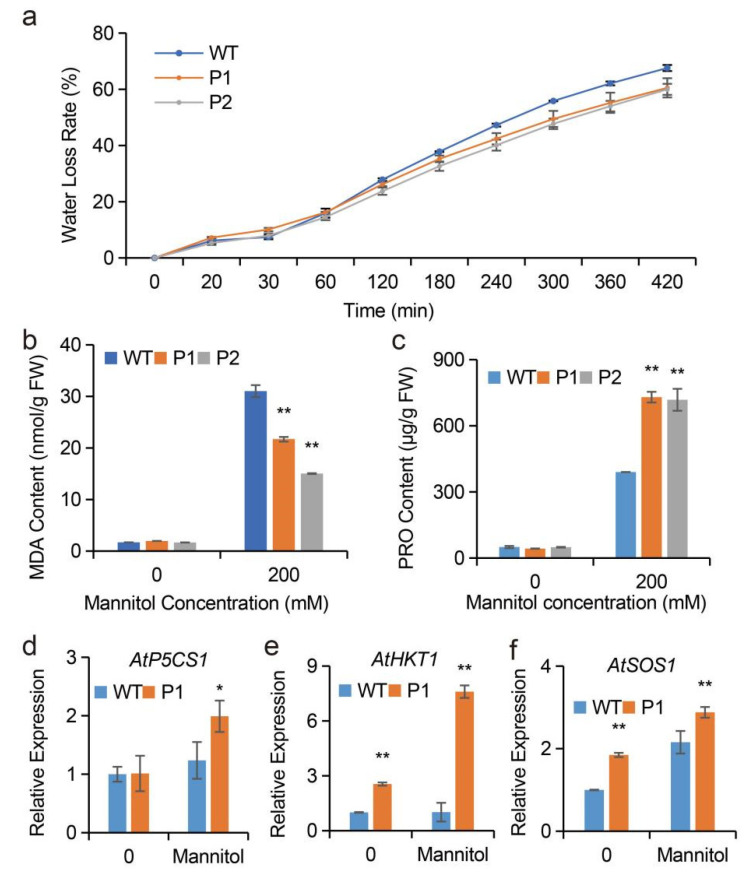
Analysis of water lose rate, MDA and PRO contents and stress-related genes under 0 or 200mM mannitol treatment. (**a**) The water lose rate. (**b**) The MDA content. (**c**) The PRO content. Expression levels of *AtP5CS1* (**d**), *AtHKT* (**e**) and *AtSOS1* (**f**) in WT and P1. (* *p* < 0.05, ** *p* < 0.01, one way ANOVA).

The tolerance to drought of WT and transgenic lines were evaluated in soil culture. Regarding transgenic *Arabidopsis* overexpressing *EuPIP1;1*, water was withheld from 40-day-old WT and transgenic lines (P1 and P2) for 30 days and then the plants were watered as normal for 7 days. The survival of transgenic tobacco plants was higher than that of the WT (Figure 7e). To further evaluate the effect of drought stress, plants were treated with 200 mM mannitol for 11 days. The results showed that the leaf growth of both transgenic and WT plants was inhibited, and the leaves of WT plants began to yellow and wilt, while the leaves of transgenic lines remained relatively healthy (Figure 7f). Due to one of the drought resistance indicators of plants, the *Arabidopsis* water loss rate was determined, and the water loss rate of transgenic lines was found to be lower than that of the WT (Figure 8a).

To further study the effects of *EuPIP1;1* overexpression on the physiology and biochemistry of transgenic *Arabidopsis*, the contents of malonaldehyde (MDA) and proline (PRO) in leaves of transgenic lines and WT plants were measured under mannitol treatment. The MDA and PRO contents of P1 and P2 were not significantly different, compared with WT, under normal conditions. However, under drought stress, the MDA contents of P1 and P2 were significantly lower than that of WT (Figure 8b), and the PRO content of transgenic lines was significantly higher than that of the WT (Figure 8c). These results indicated that the increasing tolerance to drought of transgenic *Arabidopsis* could be associated with an increase in osmotic adjustment substances and a reduction in membrane damage in transgenic plants under drought stress compared to WT.

To assess the implication of *EuPIP1;1* in the abiotic stress response pathway, we analyzed the expression levels of three genes *P5CS1* (which plays an important role in PRO biosynthesis [35]), *AtHKT1*, and *AtSOS1* (which are involved in plant ion transport [36]). The results showed that the transcriptional level of *AtP5CS1* in transgenic plants was not different from WT. However, under drought stress, *AtP5CS1* was significantly up-regulated in the transgenic plants (Figure 8d). The expression levels of *AtHKT1* and *AtSOS1* were also up-regulated in transgenic plants by mannitol treatment (Figure 8e,f). These results implied that *EuPIP1;1* could promote PRO biosynthesis by up-regulated *AtP5CS1*, and reduction of Na^+^ by up-regulated *AtHKT1* and *AtSOS1*, ultimately mitigating damage from drought stress.

### 2.6. Overexpression of EuPIP1;1 Improved Salt Tolerance in Transgenic Arabidopsis

**Figure 9:**
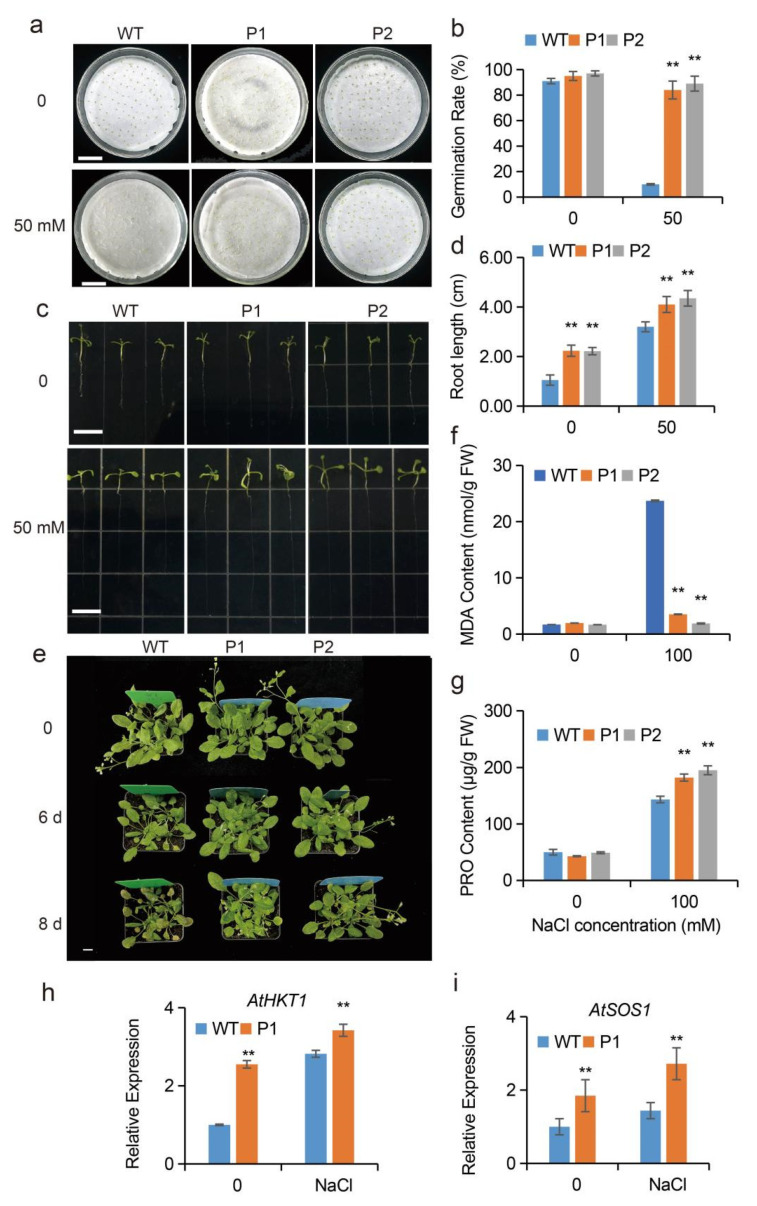
Effect of NaCl treatment on the germination and root growth of WT and transgenic lines (P1 and P2). (**a**) The germination rate of the seeds from WT and transgenic lines (P1 and P2) under 0 or 50 mM NaCl treatment. Bar = 1 cm. (**b**) The germination rate. (**c**) Phenotypes of seedlings from WT and transgenic lines (P1 and P2) treated with 0 or 50 mM NaCl treatment. Bar = 1 cm. (**d**) The length of root. (**e**) Phenotypes of seedlings from WT and transgenic lines (P1 and P2) treated with 0 or 100 mM NaCl treatment at 6 d and 8 d. Bar = 1 cm. (**f**) The MDA content. (**g**) The PRO content. Expression level of *AtHKT1* (**h**) and *AtSOS1* (**i**) in WT and P1 treated with NaCl. (** *p* < 0.01, one way ANOVA).

To study the effect of *EuPIP1;1* on the salt tolerance of transgenic *Arabidopsis*, the seeds of WT and transgenic lines (P1 and P2) were germinated on filter paper moistened with 0 or 50 mM NaCl for 7 days. Under salt stress treatment, the germination rate of P1 and P2 (by 84 and 89%, respectively) was significantly higher than that of WT (10%) (Figure 9a,b). The root length of the P1 and P2 seedlings was significantly longer (by 1.28- and 1.36-fold, respectively) than that of the WT under salt stress treatment (Figure 9c,d). To further evaluate the effect of *EuPIP1;1* on the salt tolerance of transgenic *Arabidopsis*, 40-day-old WT, P1, and P1 *Arabidopsis* seedlings were watered in pots with 100 mM NaCl. After 6 days of treatment, the leaves of the WT plants started to turn yellow, while the leaves of the transgenic plants remained green. After 8 days of treatment, the leaves of the transgenic plants began to turn yellow, while the leaves of the WT plants turned purple and withered (Figure 9e). Similar to drought stress experiments, there were no significant differences in leaf MDA and PRO contents between WT and transgenic plants under non-stress treatment. Under salt stress treatment, the MDA contents of P1 and P2 were significantly lower than that of WT (Figure 9f), and the PRO content of P1 and P2 plants was significantly higher compared with that of the WT (Figure 9g). High levels of *AtHKT1* and *AtSOS1* expression have been observed in transgenic plants treated with salt stress (Figure 9h,i). These results indicated that *EuPIP1;1* might promote root elongation, reduce membrane damage, increase the accumulation of osmotic substance, and up-regulate *AtHKT1* and *AtSOS1* to maintain ion homeostasis, ultimately improving salt resistance of transgenic *Arabidopsis*.

## 3. Discussion

*PIPs* play an important role in plant water relations. In our previous work, we proposed that overexpression of *EuPIP1;2* could improve tolerance to drought and salt in transgenic tobacco [37]. In this study, we cloned the *PIPI* subgroup gene *EuPIP1;1*, which is localized on the plasma membrane. *EuPIP1;1* is constitutively expressed in roots, stems, leaves, and fruits in *E. ulmoides*. Among the four tissues, *EuPIP1;1* and *EuPIP1;2* [37] both had the highest expression in fruits. This may be associated with the rapid development of the *E. ulmoides* fruit in August, which requires a lot of water and nutrients. However, unlike the expression of *EuPIP1;2* in roots, which was significantly higher than that in stems and leaves, there was no difference in the expression of *EuPIP1;1* in roots, stems, and leaves. Therefore, we speculated that *EuPIP1;2* was mainly involved in the transport of water in the root [37], while *EuPIP1;1* plays an important role in maintaining water transport in various tissues [38].

Gene expression studies in various plant species have shown that *PIPs* respond to environmental stimuli, and a number of *PIPs* transcripts were up-regulated under stressful conditions [39]. *ScPIP1* [40] and *ZxPIP1*;3 [14] were up-regulated in roots under drought stress and salt treatment, and *ScPIP2-1* in both below- and above-ground tissues were up-regulated under PEG and salt stress [38]. The use of transgenic plants with overexpressing or underexpressing *PIP1* also supports the importance of *PIP1* for tolerance to environmental stress. *Arabidopsis* plants expressing *ScPIP1* [40] and *CrPIP2;3* [41] had longer roots, which may have contributed to improved drought resistance. In this study, the expression level of *EuPIP1;1* was up-regulated with PEG treatment. Our results for *Arabidopsis* support the view that increased *AQPs* levels may be associated with adaptation to water stress.

Excessive accumulation of intracellular Na^+^ can not only cause ion toxicity and osmotic stress [42], but also interfere with the uptake of other ions and affect plant growth under salt stress [43]. Plants have multiple Na^+^ transport systems to circumvent Na^+^ toxicity and maintain Na^+^/K^+^ homeostasis and osmotic regulation in cells, and, thus, ensure plant growth under salt stress [42]. Research has demonstrated that the *HKT1* transporter and *SOS1* are essential for Na^+^ and K^+^ homeostasis in plants [36]. Up-regulation of *HKT1* and *SOS1* contributes to Na^+^ efflux and K^+^ absorption; thus, maintaining and reconstructing cellular ion homeostasis and alleviating plant damage under drought and salt stress [42]. Studies have shown that overexpression of *PIPs* can increase *HKT1* and *SOS1* to improve tolerance to salt stress in transgenic plants [44]. In our study, *HKT1* and *SOS1* were up-regulated in transgenic *Arabidopsis*, indicating that *EuPIP1;1* could promote the expression of the two genes to maintain Na^+^/K^+^ homeostasis and ensure plant growth under drought and salt stress.

The production of reactive oxygen species (ROS) in plants is a defense response to various stresses, while excessive amounts can cause damage [45]. As an osmotic agent and free radical scavenger, PRO can accumulate to protect cells from damage under abiotic stresses [35]. MDA is commonly used as a marker for ROS-mediated damage [46]. *ScPIP1* [40], *ZxPIP1*;3 [14], *MaPIP1;1* [47], *EuPIP1;2* [37] etc., can promote the accumulation of PRO and reduce the production of MDA in transgenic plants, thereby keeping transgenic plants in a relatively healthy physiological state and improving tolerance to drought and salt. In our study, under normal conditions, the contents of PRO and MDA were not different between WT and transgenic plants. However, under drought and salt treatment, the PRO contents of P1 and P2 were significantly higher than those of WT, and the MDA content was lower than that of WT. It was suggested that *EuPIP1;1* can maintain the stability of the cell membrane by increasing the accumulation of osmotic substances and reducing the content of membrane damage, thus improving the resistance to stress of *Arabidopsis*.

The higher expression of *AQPs* in growth, compared to non-growing, tissues is often associated with higher cell hydraulic conductivity (Lp) in growing tissue [48]. *PIPs* play an important role in regulating rapid transmembrane water flow during plant growth and are believed to be directly involved in cell growth [49]. In this study, the transgenic lines assessed both showed significant increase in stomatal size and cell area. This might be associated with overexpression of *EuPIP1;1*, which enhanced the water uptake in transgenic *Arabidopsis* and thereby promoted cell growth. Studies have shown that overexpression of *PtoPIP1;1* in *Arabidopsis* could accelerate cell growth in leaves and roots and promote bolting and flowering by up-regulation of growth- and flower-related genes [49]. Similarly, in our study, the results showed that *EuPIP1;1* accelerated the growth of leaves and roots, and promoted bolting and flowering in *Arabidopsis*. The expression level of the plant growth and development-related genes, *AtPIF4* and *AtTCP14*, and the flowering related genes, *AtCRY1*, *AtCRY2*, *AtFCA* and *AtFT*, were significantly up-regulated in transgenic plants. These results indicated that overexpression of *EuPIP1;1* could promote leaf development by expending cell size in transgenic *Arabidopsis* and up-regulating the transcriptional level of plant growth- and flowering-related genes, ultimately promoting the growth of the plant and accelerating bolting and flowering.

In conclusion, we found that *EuPIP1;1* was induced by drought treatment and played an important role in maintaining water transport in various tissues. Overexpression of the *EuPIP1;1* accelerated flowering and bolting, and promoted leaf development by expending cell size. The expression levels of flowering-related genes (*AtCRY1*, *AtCRY2* and *AtFCA*) and hub genes in the regulatory networks underlying floral timing (*AtFT*) were significantly up-regulated in transgenic *Arabidopsis*. Moreover, overexpression of *EuPIP1;1* enhanced the drought and salt tolerance in transgenic *Arabidopsis* by increasing accumulation of osmotic substances, reducing membrane damage and maintaining ion homeostasis. These results showed that *EuPIP1;1* has potential use in improving plant stress tolerance.

## 4. Materials and Methods

### 4.1. Plant Materials and Growth Conditions

Ten-year-old *E. ulmoides* female plants from the Guizhou local variety, cultured in the Transgenic Plant Demonstration Base of Guizhou University, Guiyang (106°40′ E, 26°24′ N), were selected for gene cloning and tissue expression analysis [50]. Three-year-old plants, which germinated from the seeds of gene cloning tree, were used for drought treatment. *N. benthamiana* was used as the recipient for visualization of the subcellular localization of the protein. *Arabidopsis* ‘Columbia-0’ was used as wild-type (WT) and all transgenic lines were generated in the background of ‘Columbia-0’ in this study. All transgenic lines and WT *Arabidopsis* plants were grown at 23 °C in 16 h light/8 h dark cycles, the light intensities are 10,000 lux.

### 4.2. Full cDNA Cloning and Bioinformatic Analysis of EuPIP1;1

For gene cloning, total RNA was extracted from leaves following the method of Gambino, et al. [51]. Full-length cDNA sequence of *EuPIP1;1* was cloned by the RACE method using the SuperScript II RT Kit (Invitrogen, Carlsbad, CA, USA), according to the manufacturer’s instructions. The primers used for RACE are listed in Table S2.

For phylogenetic analysis, a multiple alignment was performed by DNAMAN 6.0 software (Lynnon Biosoft, QC, Canada), the phylogenetic tree was constructed using MEGA 11 software (Mega Limited, Auckland, New Zealand), and the maximum likelihood method with 1000 bootstrap replicates was used to determine the phylogeny in the neighborhood joining tree (NJ). The three-dimensional structure of the protein was modeled using the SWISS-MODEL server (https://swissmodel.expasy.org/ (accessed on 22 March 2022)).

### 4.3. Subcellular Localization of Protein

To construct the protein localization vector pCambia-35S-*EuPIP1;1*::GFP, the *EuPIP1;1* full-length coding sequence without stop codon fused with GFP was inserted into the binary vector pCambia1300. Then, the vector was introduced into the *Agrobacterium tumefaciens* strain EHA105. Transient expression was performed using the leaves of *N. benthamiana* according to the method of Li [52]. The GFP fluorescence signal was observed with a laser confocal microscope (Leica TCS SP8 STED, Wetzlar, Lahn-Dill-Kreis, Germany) under excitation at 488 nm.

### 4.4. Quantitative Real-Time PCR

For tissue-specific expression, total RNA was extracted from leaves, roots, stems and fruit of 10-year-old *E. ulmoides* female plants using TRIzol reagent according to the method of Chen, et al. [37]. The RNA quality was detected by the absorbance at A260/A280 and A260/A230 (Table S1). Reverse transcription and quantitative RT-PCR (qRT-PCR) analysis were performed, as described by Zeng and Zhao [53]. Each test was repeated three times.

Three-year-old *E. ulmoides* plants were treated with 20% PEG6000 [40], and then the total RNA was extracted at 0, 1, 2, 6, 24, and 48 h from leaves using E.Z.N.A.™ Plant RNA Kit (Omega, Norcross, GA, USA). cDNA was reversed with the StarScript II First-stand cDNA Synthesis Mix with gDNA Remover Kit (GenStar, Beijing, China). Using the 2 × RealStar Green Fast Mixture (GenStar, Beijing, China) for qRT-PCR according to the manufacturers’ protocols (two-step approach). All the data were calculated and analyzed from three independent samples. The *EuActin* gene was used as the internal control gene [54]. The 2^−∆∆Ct^ method was used to determine the relative expression level [55]. All primers for qRT-PCR were listed in Table S2.

### 4.5. Overexpression Vector Construction and Plant Transformation

**Figure 10:**
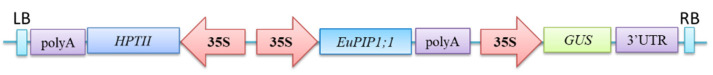
The T-DNA region of pCambia1301-35S-*EuPIP1;1* vector.

The full-length coding sequence of *EuPIP1;1* was amplified by chemical synthesis and inserted into the pCambia1301 vector (Figure 10). The vector was introduced into the *Agrobacterium tumefaciens* strain EHA105, and then transformed into *Arabidopsis* via the floral dipping method. The seeds were then selected with hygromycin (50 mg/L), and the generation of T_3_ was used for subsequent experiments.

### 4.6. Drought and Salt Tolerance Analysis of Transgenic Plants

For the analysis of germination, *Arabidopsis* seeds of two transgenic lines P1, P2, and WT were seeded on filter paper moistened with 0, or 200, mM mannitol and incubated for 7 days (23 °C, 16 h light/8 h dark). For root length assays, seeds were treated with 75% ethanol for 30 s, then sterilized with 10% NaClO solution for 5 min, and rinsed with sterile water 5 times. The seeds were cultured on half-strength Murashige and Skoog (1/2 MS) medium supplemented with 0, 200 mM mannitol or 50 mM NaCl for 17 days (23 °C, 16 h light/8 h dark), and then the lengths of the roots were measured.

The 40-day-old seedlings were treated without watering for 30 days, and then the plants were re-watered as normal for 7 d. The 50-day-old seedlings from WT and transgenic lines were used for water loss analysis, following the method described by Aharoni, et al. [56]. The percentage of water loss was calculated with the formula: water loss rate (%) = (FW − DW)/FW × 100. Leaves from 60-day-old *Arabidopsis* were treated with clear nail polish, as described previously [57]. Then, stomatal size (µm^2^) was calculated using ImageJ 1.49 (National Institute of Health (NIH), Bethesda, USA) as the guard cell length × guard cell pair width from images collected at ×40 magnification [58]. Leaves from 60-day-old *Arabidopsis* were fixed in formalin-acetic acid-alcohol (FAA) and then cleared in chloral hydrate solution [49], and images were collected at ×40 magnification.

### 4.7. MDA and PRO Detection

For MDA and PRO detection, the 40-day-old *Arabidopsis* seedlings were watered with or without 200 mM mannitol or 100 mM NaCl for 8 d every 2 days. The leaves were then collected to determine the contents of MDA and PRO. The PRO and MDA content were determined using the Ninhydrin Reaction PRO Detection Kits (Keming, Suzhou, China) and Thiobarbital Acid (TBA) Reaction MDA Detection Kits (Keming, Suzhou, China), according to the manufacturer’s instructions, PRO content (μg/g FW) = 38.4 × (A520 + 0.0021)/W, MDA content (nmol/g FW) = 51.6 × (A532 − A600)/W. Each test was repeated three times.

### 4.8. The Expression Analysis of Growth- and Stress-Related Genes

Total RNA was extracted from leaves of 50-day-old seedling cultured under normal conditions using E.Z.N.A.™ Plant RNA Kit (Omega, Norcross, GA, USA). The cDNA was reversed with the StarScript II First-stand cDNA Synthesis Mix with gDNA Remover Kit (GenStar, Beijing, China). The expression level of *EuPIP1;1* in transgenic plants was evaluated. The level of transcripts of development-related genes (*AtGIF2*, *AtKUP4*, *AtLBD18*, *AtPIF4*, and *AtTCA14*) and flowering-related genes (*AtCRY1*, *AtCRY2*, *AtFCA* and *AtFT*) were evaluated. *AtActin2* was used as the internal control.

The 40-day-old *Arabidopsis* seedlings were watered with or without 100 mM NaCl or 200 mM mannitol for 9 d every 2 days. Leaves from each treatment were collected and total RNA was extracted. The level of *AtHKT1*, *AtP5CS1* and *AtSOS1* were evaluated.

All the data were calculated and analyzed from three independent samples. All primers for qRT-PCR were listed in Table S2.

### 4.9. Statistical Analysis

All data shown as mean ± standard error of means was analyzed using one-way ANOVA, followed by Duncan’s test, using IBM SPSS Statistics 25 software (IBM Corporation, Chicago, IL, USA). Statistically significant mean values were indicated as * (*p* < 0.05) or ** (*p* < 0.01).

## Data Availability

Not applicable.

## Supplementary Materials

The following supporting information can be downloaded at: https://www.mdpi.com/article/10.3390/ijms231911794/s1.
