# Supplementary material for: Overexpression of the Eucommia ulmoides Aquaporin, EuPIP1;1, Promotes Leaf Growth, Flowering and Bolting, and Stress Tolerance in Arabidopsis
Source: Int J Mol Sci. 2022 Oct 4;23(19):11794. doi: 10.3390/ijms231911794 (PMC9570377; doi:10.3390/ijms231911794)
Supplement: Supplementary file 1 [file ijms-23-11794-s001.zip › ijms-1859292-supplementary.pdf]

Supplementary Materials

5’UTR

cgagcggggcggagcttgcttctggagtcttcaaatatagagagagaaaggtcgaattctagagagagtgtgaagATGGAGGGTAAG
GAAGAGGATGTGAAGCTCGGAGCGAACAAATTTCCCGAGAGGCAGCCGTTGGGAACTGCGGCGCAGACG
GACAAGGACTACAAGGAGCCACCGCCGGCGGGCCTGTTCGAGCGGGAGGAGCTGACTTCATGGTCGTTTT
ACAGGGCCGGAATCGCGGAGTTCATGGCGACTTTCCTGTTCTTGTACATCACGGTCTTGACGGTGATGGGT
GTTAACAGAGCGCCGTCCAAGTGCGCCTCCGTCGGCATCCAAGGCATCGCTTGGGCCTTCGGCGGTATGAT
CTTTGCCCTCGTCTACTGCACCGCCGGTATCTCAGGAGGACACATTAACCCGGCGGTGACCTTCGGTTTGTT
CCTGGCCAGAAAACTATCGCTAACCAGGGCGATCTTCTACATCGTGATGCAATGCCTTGGCGCAATTTGCGG
TGCCGGCGTGGTGAAGGGGTTCCAACAGTCGGAATTCCAGATGACCGGTGGTGGAGCCAACTTTGTTGCC
CCTGGCTACACCAAGGGTGACGGCCTCGGTGCGGAGATCGTCGGTACCTTTGTTCTTGTCTACACCGTTTTC
TCCGCCACTGATGCCAAGAGAAGCGCCAGAGACTCTCATGTCCCTATTTTGGCTCCTCTCCCCATCGGGTTTG
CGGTGTTCTTGGTCCATTTGGCGACAATCCCCATCACTGGAACCGGCATCAACCCTGCCCGGAGTCTCGGAG
CTGCCATCATTTACAATAAAGATCATGCCTGGGACGACCACTGGATATTCTGGGTTGGACCCCTCATTGGAGC
TGCGCTTGCTGCTTTATACCACCAGATAGTCATCCGAGCAATTCCTTTCAAGAG

3’UTR

CAGGGCTTAAaaaacccttattttctttcaagtccttttcttttttcttcttttttatttggtccttgtttttcttgttcttgtggtgttgctctgtgta
aattatgataattaggtttgttttgtgtattttaaggatgttggatgaatgaaatgaacaaattattattgttaacaaaaaaaaaaaaaaaaaa
aaaaaaaaaa

polyA

Figure S1. Coding sequence of *EuPIP1;1*.

Table S1. The absorbance of OD(A260/A280) and OD(A260/A230) of the RNA.

Sample	OD(A260/A280)	OD(A260/A230)
RNA extracted form Leaves of *E. ulmoides* PEG treatment for investigate the expression mode of *EuPIP1;1* in *E. ulmoides* under PEG stress		
0h 1	1.92	2.51
0h 2	1.97	2.09
0h 3	1.93	1.98
1 h 1	2.01	2.21
1 h 2	1.96	2.23
1 h 3	1.88	1.82
2 h 1	2.0	2.34
2 h 2	2.03	2.10
2 h 3	1.91	1.80
4 h 1	1.98	2.21
4 h 2	1.91	1.75
4 h 3	1.87	2.43
6 h 1	2.05	2.45
6 h 2	1.93	1.99
6 h 3	1.84	1.89
24 h 1	2.06	2.43
24 h 2	1.96	2.01

24 h 3	1.95	2.51
48 h 1	1.97	2.52
48 h 2	1.96	1.91
48 h 3	1.90	1.93
RNA extracted from *E. ulmoides* for tissue-specific expression		
root1	1.98	2.42
root2	1.85	2.23
root3	1.96	2.46
stem1	1.87	2.12
stem2	1.99	2.17
stem3	1.86	2.25
leaf1	2.01	2.28
leaf2	1.99	2.45
leaf3	1.87	2.23
fruit1	1.88	2.25
fruit2	1.92	2.41
fruit3	1.99	2.3
RNA extracted from leaves of *Arabidopsis* for analysis the expression level of growth-related genes and the expression level of *EuPIP1;1*		
WT 1	2.01	2.54
WT 2	1.85	1.92
WT 3	2.01	2.50
P1 1	2.01	2.46
P1 2	1.89	2.39
P1 3	1.99	2.38
P2 1	1.89	2.45
P2 2	1.94	2.42
P2 3	2.01	2.25
RNA extracted from leaves of *Arabidopsis* for analysis the expression level of stress-related genes		
WT 1	1.96	2.35
WT 2	2.01	2.45
WT 3	1.90	1.93
P1 1	1.94	2.23
P1 2	1.83	2.36
P1 3	2.0	1.98
WT 1 (drought)	1.86	2.25
WT 2 (drought)	1.93	2.11
WT 3 (drought)	1.94	2.50
P1 1 (drought)	1.98	2.28
P1 2 (drought)	1.87	1.98
P1 3 (drought)	1.91	2.34
WT 1 (salt)	1.99	2.27

WT 2 (salt)	1.86	2.42
WT 3 (salt)	1.90	2.07
P1 1 (salt)	1.93	2.31
P1 2 (salt)	1.96	2.01
P1 3 (salt)	1.96	1.99

Table S2. Primers used in the study.

Primer	Gene ID	Sequence 5′ to 3′	Purpose
EuPIP-GSP1		GGTGGCTCCTTGTAGT	5′RACE
EuPIP-GSP2		TGCGCCGCAGTTCCCAA	
EuPIP-GSP3		TGCCTCTCGGGAAATTTG	
EuPIPC3-1		CCCCATCGGGTTTGCGGTGTTCTT	3′RACE
EuPIPC3-2		CCGGAGTCTCGGAGCTGCCATCAT	
EuPIP-F		GTCGAATTCTAGAGAGAG	Full cDNA sequence
EuPIP-R		TTTAAGCCCTGCTCTTGA	
qEuPIP-F		TCCAGATGACCGGTGGTG	Realtime PCR
qEuPIP-R		AGTGGCGGAGAAAACGGT	
EuActin-F		TTGTTAGCAACTGGGATGATATGG	Realtime PCR
EuActin-R		CAGGGTGTTCTTCAGGAGCAA	
EuPIPJ-F		TCCAGATGACCGGTGGTG	Transgenic plant detection
EuPIPJ-R		AGTGGCGGAGAAAACGGT	
AtACT2-F	AT3G18780	AAGTCTTGTTCCAGCCCTCG	Realtime PCR
AtACT2-R		TCTGCTGGAATGTGCTGAGG	
AtGIF2-F	AT1G01160	GATGCTTGGTGGGTCAGGAA	
AtGIF2-R		GCGCACTCAGCAAGTTTACC	
AtKUP4-F	AT4G23640	TTACCCGTGTCTGGTGCTTC	
AtKUP4-R		TGACAATCGAGAACGTGGCA	
AtLBD18-F	AT2G45420	TCCTCCCCCTCTTGCCATAA	
AtLBD18-R		CACCGTATTGGTGACGTGGA	
AtPIF4-F	AT2G43010	CGGAGTTCAACCTCAGCAGT	
AtPIF4-R		CCATCGGCTGCATCTGAGTC	

AtTCP14-F	AT3G47620	TCCTTCTCATTTCCGCTCCG
AtTCP14-R		TAGGTGCACGTCCCTGTAGA
AtP5CS1-F	AT2G39800	ATCCCTGTGCTAGGTCATGC
AtP5CS1-R		CTATGCGCTTTGCCATATCCG
AtHKT1-F	AT4G10310	TCTTCTTGGAGTGACGGTGC
AtHKT1-R		TAGTTTCTCCGGTGTGTCGC
AtSOS1-F	AT2G01980	TCATCTCCCGCCGCATTATC
AtSOS1-R		CTTGAGCTACGTGGTGGTGT
AtCRY1-F	AT4G08920	TGCTGCGTTTTGGGAGAGAT
AtCRY1-R		GCACTATCGGCTTTTCTGCG
AtCRY2-F	AT1G04400	GATGCCAATAACTGCAGCGG
AtCRY2-R		GGCACACTGGAAAACGTGTC
AtFCA-F	AT4G16280	AGCCTATGGGTCCTCACCAT
AtFCA-R		GAAGGAGGATACTTCGCGGG
AtFT-F	AT1G65480	ATGTGTAGAGGGTTCATGCC
AtFT-R		TCCTGAGGTCTTCTCCACCAA
